# Barriers and Facilitators of Implementing Workplace Interventions Supporting Young Workers’ Safety, Work Environment and Health: A Scoping Review of Qualitative and Mixed-Method Studies

**DOI:** 10.1007/s10926-025-10313-3

**Published:** 2025-08-23

**Authors:** Emil Sundstrup, Johnny Dyreborg, Anders Dreyer Frost, Karina G. V. Seeberg, Lars Louis Andersen, Thomas Clausen

**Affiliations:** 1 National Research Centre for the Working Environment, Lersø Parkallé 105, 2100 Copenhagen, Denmark; 2 Research Unit of Physical Activity and Health in Working Life, Department of Sports Science and Clinical Biomechanics, University of Southern Denmark, Odense, Denmark; 3 Section of Social Medicine, Department of Public Health, University of Copenhagen, Copenhagen, Denmark

**Keywords:** Intervention, Workers, Injuries, Accidents, Work environment, Implementation, Adolescent, Young adult

## Abstract

**Purpose:**

Young workers face higher risks of workplace accidents, mental health issues, and physical strain. This scoping review aims to identify and summarize available research literature on barriers and facilitators to implementing workplace interventions to support young workers’ safety, work environment and health.

**Methods:**

We conducted a comprehensive search in bibliographic databases including PubMed, Web of Science and PsycInfo for articles published from 2007–2022. The PICO strategy guided the assessment of relevant studies and the bibliographical search for qualitative and mixed-method studies on interventions where (1) participants were young workers (mean age 15–29 years), (2) interventions were initiated and/or carried out at the workplace, and (3) barriers or facilitators to the implementation of interventions to support young workers’ safety, work environment and health were described. We employed an iterative process to identify general thematic categories in the data.

**Results:**

In total, 11 relevant studies were identified. Seven primary themes related to facilitators and barriers emerged from our analysis. Facilitators: fit the organizational context, organizational support, balance between efforts and gains, employee motivation, and employee involvement; Barriers: high workload and time pressure, shift work and irregular working hours.

**Conclusion:**

There is a lack of intervention studies on young workers focusing on factors for implementation. The studies we find, reveal several factors to be aware of when designing and implementing interventions to support young workers’ safety, work environment and health. The results emphasize a need for studies evaluating factors of importance for the successful implementation of workplace-based interventions among young workers.

**Review registration:**

PROSPERO CRD42022324299 (https://www.crd.york.ac.uk/prospero/display_record.php?ID=CRD42022324299).

**Supplementary Information:**

The online version contains supplementary material available at 10.1007/s10926-025-10313-3.

## Background

The work environment has an important influence on health and well-being of workers [1]. Previous research shows that young workers are faced with adverse working conditions in terms of exposure to occupational accidents, poor safety climate, physically demanding work tasks and chemical substances in the work environment [2–5]. Moreover, the research also shows a higher prevalence of depression and anxiety symptoms among young workers in Denmark [3], and research suggests that these mental health conditions are associated with factors in the psychosocial work environment [2, 6]. Taken together, these findings indicate that young workers are confronted with significant challenges in their work environment, which on the one hand may compromise their immediate well-being, and on the other hand the long-term sustainability of the work-lives of the young workers. This vulnerability of young workers has led to greater global awareness of their occupational health and safety, highlighting the urgent need for early preventive measures in the workplace.

Several characteristics of young workers suggest that implementing workplace interventions for this group might present unique challenges. First, young workers typically have limited work experience and less professional networks, which may influence how they engage with and respond to workplace interventions [7, 8]. Second, young workers often may have lower awareness of occupational safety and health (OSH) [9, 10]. Third, young workers often have more precarious jobs and less influence at the workplace, which could make them less likely to be involved in workplace interventions [11, 12].

Since young workers are confronted with several challenges to their work environment and health they may need a special attention in the OSH efforts at the workplace. Therefore, it is important to identify and summarize barriers and facilitators affecting the implementation of interventions targeting this vulnerable group of young workers. Understanding these factors for implementation—whether they reflect more general barriers/facilitators or emphasize more unique barriers/facilitators for young workers—is essential for designing and implementing interventions that effectively improve young employees work environment, safety, and health. To our knowledge, no previous reviews have identified and summarized factors of importance for implementing occupational health interventions aiming at young workers. Such knowledge will give relevant practitioners (e.g., OSH practitioners) a stronger knowledge base to act on and may assist them in successfully implementing the best solutions applicable for improving work environment, safety and health for this vulnerable group of workers.

In a previous systematic review, we examined the literature to identify effective workplace interventions aimed at supporting young workers’ work environment, safety and health [13]. That review identified 33 studies focusing on interventions targeting young workers (i.e., mean age of 15 to 29 years). However, the evidence synthesis revealed that the effectiveness of these interventions was limited, inconsistent, or insufficient for improving working conditions and workplace safety, and these findings may reflect challenges in implementing such interventions. This implies that the processes by which workplace efforts targeting improvements in the work environment, safety and health of young workers are of pivotal importance for the effectiveness of these interventions in concrete workplace settings. Systematic reviews on workplace interventions—not with a specific focus on young workers—indicate that the following factors are important for the successful implementation of interventions in specific workplace settings:

- a participatory approach towards developing and implementing improvements in work environment, safety and health [14];
- unequivocal support from the top and middle management in the workplace [14–16];
- adequate resources in terms of e.g., time and staff [14, 15];
- a fit between the organization and the proposed intervention [14];
- clear communication about the intervention [14]; and
- worker support and motivation to contribute to the successful implementation of the intervention [16].

Despite these insights on more general factors of importance, little is known about the specific barriers and facilitators affecting the implementation of interventions targeting young workers. What are the facilitators and barriers to successful implementation that should be considered when designing and implementing interventions for this group of workers? Are there specific factors that must be taken into account when deploying workplace interventions targeting young employees, or are these factors more general? Understanding these factors is essential for designing and conducting interventions that effectively improve the work environment, safety, and health of this group. It could be speculated, that young workers may have limited experience in the work environment and lower awareness, knowledge, or interest in occupational health and safety, which could influence how interventions are received and implemented. Therefore, specific implementation factors could require greater emphasis when implementing workplace interventions for young workers.

Therefore, this scoping review aims to identify and summarize available research literature on barriers and facilitators to implementing workplace interventions to support the work environment, safety and health of young workers. In this study, we define a barrier as any condition that may impede the delivery or implementation of an intervention, and we similarly define a facilitator as any condition that furthers the delivery or implementation of an intervention.

The knowledge that is generated in this scoping review might be highly relevant for practice for several reasons. First, the results provide an evidence-base for designing workplace interventions that target the specific needs of young workers, thereby supporting a conducive psychosocial and ergonomic work environment [17]. Moreover, the findings from this scoping review may contribute with knowledge on how to foster critical awareness among young workers about their work environment [18]. Giving young workers such awareness is essential in ensuring that workplace safety and health are recognized as important. By highlighting the challenges young workers face, this review underscores the need to strengthen their engagement with occupational health and safety. The creation of this type of awareness may also be an instrumental part in the promotion of attention towards the work environment that may prove an important prerequisite for long, safe and healthy work-lives for young workers.

## Material and Methods

### Study Design

We conducted a scoping review of qualitative and mixed methods studies, identified through a comprehensive search in PubMed, PsycInfo and Web of Science Core Collection.

The reporting of the study follows the PRISMA-Scr guidelines (The reporting Preferred Reporting Items for Systematic reviews and Meta-Analyses extension for Scoping Reviews; https://www.prisma-statement.org/scoping).

The present review is a part of a larger review project that has been collectively registered in the International Prospective Register of Systematic Reviews (PROSPERO; CRD42022324299): https://www.crd.york.ac.uk/prospero/display_record.php?ID=CRD42022324299.

### Eligibility Criteria

Table 1 displays the eligibility criteria based on the PICO strategy used in this review. The PICO strategy guided our assessment of study relevance and the bibliographical search for studies that focused on workers or the transition to work. We used the following inclusion criteria: (1) participants were young workers with a mean age of 15 to 29 years; (2) interventions were workplace-based; and (3) describes factors that may act as barriers or facilitators in the implementation of interventions to support young workers’ safety, work environment and health.

**Table 1:** Illustration of the PICO used for the present review

P	Population	Young workers (mean age: 15–29 years)
I	Intervention	Initiated by the workplace, supported by the workplace and/or carried out at the workplace (i.e., workplace-based)
C	Comparison	No comparator intervention is required
O	Outcome	Describe factors that may act as barriers or facilitators in the implementation of interventions to support young workers’ safety, work environment and health

We did not exclude specific study designs as long as they adhered to the inclusion criteria mentioned above. Thus, we included any study that described barriers or facilitators to the implementation of a workplace OSH intervention among young workers. Studies could report a qualitative study (qualitative methods of data collection and analysis, e.g., interviews or observations), a quantitative study (quantitative method of data collection e.g., controlled trials) or a mixed methods study (combines qualitative and quantitative methods of data collection and analysis). The publication language of the included studies was English or Scandinavian. The review focuses on primary OSH interventions that deliberately aim to support safety, work environment, and health and reduce the frequency or severity of injuries or well-being at work [13, 19]. Therefore, only studies reporting an intervention directed at the workplace (i.e., workplace-based interventions) are included. An intervention could be initiated at the workplace by the employer, employees, or externally by public authorities, social partners, or other stakeholders.

### Search Strategy

The comprehensive search was conducted across multiple bibliographic databases, including PubMed (including the database 'MEDLINE'), Web of Science Core Collection (including the databases 'Science Citation Index Expanded', 'Social Sciences Citation Index', and 'Arts & Humanities Citation Index'), and PsycInfo via OVID. To perform the search, the following three main components were combined: (1) young workers (mean age: 15–29 years); AND (2) workplace intervention AND (3) date (published from 2007 to 2022. The search strategy for each database can be found in Supplementary Material 1. The search strategy was identical to the search reported in our previous systematic review with the aim of investigating the effectiveness of workplace interventions to support young workers’ work environment, safety and health [13]. More details about the search can be found in that publication [13].

### Assessment of Relevance and Inclusion

**Fig. 1:**
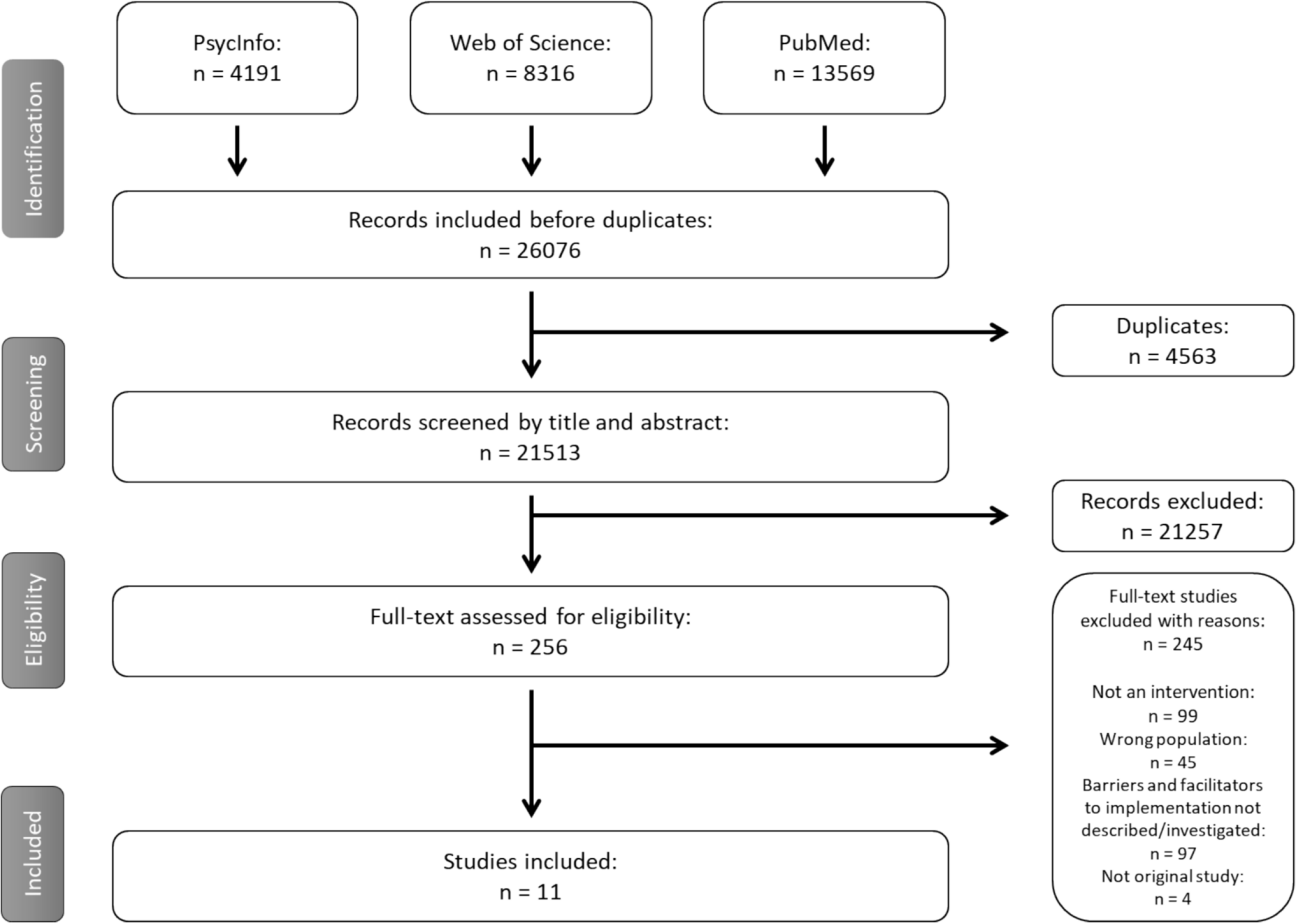
Flow chart

The study selection process is summarised in the flow chart presented in Fig. 1. In short, we collected all relevant studies, from PubMed, Web of Science Core Collection, and PsycInfo, using EndNote X9. By the use of Covidence [20], which is a screening and data extraction tool for conducting systematic reviews. The abstracts of potential studies were then independently screened by pairs of review authors, with disagreements resolved through discussion involving a third review author. Full-text publications of those studies deemed relevant by the abstract screening were also assessed similarly. Only studies adhering to the eligibility criteria presented in the PICO (Table 1) were included in the review.

#### Data Extraction and Thematic Coding/Analysis

Two authors (A.D.F and T.C.) independently extracted data from included studies into a piloted Word document. We extracted data on the following: first author, year of publication, study design, geographical setting (country), study population, data collection methods, type of intervention, barriers to implementation, and facilitators for implementation. Discrepancies were resolved through discussion.

We analysed the studies using the framework of thematic analysis offered by Braun and Clarke [21]. By following the six steps described in thematic analysis, two of the authors (A.D.F. and T.C.) familiarized themselves with the data by thoroughly reading all papers included in the review (step 1). Subsequently, the Results and Discussion sections of the 11 studies were coded for information on barriers and facilitators for implementation of interventions (step 2). From these codes, we inductively identified, defined and named themes in the data. The coded papers that were included in the review were jointly analyzed by A.D.F. and T.C. (step 3) and the identified themes were discussed and agreed upon by all authors (step 4 and 5) in an iterative process where steps 2 to 5 were revisited until the authors were in agreement of the seven identified themes. The seven themes describe facilitators and barriers related to the implementation of interventions towards young workers in the thematic analysis. The identified themes are presented below (step 6) in the Results section and Tables 2 and 3.

**Table 2:** Study characteristics of included studies

Study (author(s), year)	Objectives	Study type	Country	Population	Data collection methods	Themes related to barriers and facilitators to implementation
Albertsen et al. 2021 [18]	To evaluate the implementation of an intervention to establish H&S groups for young workers in Danish supermarkets	Mixed methods	Denmark	Young workers and managers from 10 supermarkets	Questionnaires and various qualitative data sources	Organizational support to the intervention Employee motivation Employee involvement
Deady et al. 2020 [22]	To (1) qualitatively evaluate the HeadGear app within an apprentice population; and (2) evaluate the usability, acceptability, feasibility, and preliminary efficacy of a modified version of the app (HeadGear Apprentice), designed to reduce depressive symptoms in an apprentice working population	Mixed methods	Australia	Apprentices (Commercial cookery/hospitality, Electronics, Construction trade (plumbing, bricklaying, carpentry, electrician), Other)	Questionnaires and interviews	Fit between the intervention and the organizational context Employee involvement
Fainstad et al. 2018 [23]	To increase understanding of “how and why” the Prepare to ADAPT Framework affects the learners’ feedback experiences and their ability to identify areas of their performance to improve upon. The central research question is: what are learners’ perceptions of engaging with the Prepare to ADAPT Framework, and was the framework useful for improving the feedback process?	Qualitative	USA	Medical Trainees	Interviews	Fit between the intervention and the organizational context
Fish et al. 2022 [24]	To identify the evolution of medical education within the workplace and to explore the effectiveness of the Wrap Around intervention as perceived by staff	Qualitative	England	Junior doctors	Interviews	Fit between the intervention and the organizational context Organizational support to the intervention Employee motivation High workload and time pressure
Grüne et al. 2022 [25]	To evaluate co-created multi-component physical activity interventions in vocational education and training in nursing care and automotive mechatronics regarding (1) their sustainable implementation at the institutional level and (2) the effectiveness of single intervention components at the individual level	Mixed methods	Germany	VET institutions in the nursing care sector (a school) and in the automotive mechatronics sector (a company)	Questionnaire survey and semi-structured interviews	Fit between the intervention and the organizational context Organizational support to the intervention Employee motivation High workload and time pressure
Gummesson et al. 2016 [26]	To analyze the effect of QR codes that link to Picture Mix EXposure (PIMEX) videos by analyzing attitudes to this safety training method and safety in student responses	Mixed methods	Sweden	Upper secondary school programs directed towards the carpentry industry and furniture sector	Interviews	Employee motivation
Kang et al. 2019 [27]	To assess the impacts and feasibility of a mindfulness-based intervention program as an occupational intervention in a metropolitan hospital	Mixed methods	Australia	Psychiatry trainees	Questionnaires and qualitative data through an open-ended feedback survey	Fit between the intervention and the organizational context High workload and time pressure
Luton et al. 2021 [28]	To assess the influence of a novel enhanced stress-resilience training (ESRT) course delivered at the start of core surgical training in a single UK statutory education body	Mixed methods	United Kingdom	Surgical trainees	Questionnaires and interviews	Effort–gain balance Shift work and irregular working hours
Pakenham et al. 2013 [29]	To explore: (1) CPTs’ perceptions of a previously evaluated Acceptance and Commitment Therapy (ACT) stress management intervention; and (2) their satisfaction with clinical training and suggested programme changes	Mixed methods	Australia	Postgraduate clinical psychology students	Questionnaires and qualitative data through open-ended questions	High workload and time pressure
Rich et al. 2020 [30]	To assess the feasibility, acceptability and impact of a novel intervention to reduce burnout and improve wellbeing	Mixed methods	England	Doctors-in-training (post-graduate medical training)	Questionnaires and interviews	Effort–gain balance Employee motivation High workload and time pressure Shift work and irregular working hours
Sørø et al. 2021 [31]	To explore the influences of an inter-professional preceptor-team intervention (IPPT) on inter-professional collaboration, preceptors’ role, confidence, and motivation to precept health care students (nursing, physio-therapy) and apprentices in a Norwegian nursing home	Qualitative	Norway	Preceptors in one Norwegian nursing home with four wards offering clinical learning to nursing and physiotherapy students, nursing apprentices and pupils in health care and social work	Focus group interviews	Fit between the intervention and the organizational context Organizational support to the intervention High workload and time pressure Shift work and irregular working hours

**Table 3:** Overview of the seven themes identified related to barriers and facilitators for implementation

Facilitators	
Fit between the intervention and the organizational context	Fit between intervention and the workplace [24, 25, 27] Perceived relevance of intervention [22, 23, 31]
Organizational support to the intervention	Support to intervention from top- and middle-level management [18, 24, 25, 31] Support to intervention from OSH-representatives and union representatives [18]
Effort–gain balance	Intervention activities must be accessible [28] Intervention activities must not take too much time [30]
Employee motivation	The motivation of employees is an important prerequisite in the implementation of interventions [18, 24–26, 30]
Employee involvement	Involvement of employees in identifying, designing and implementing interventions [18, 22]

Barriers	
High workload and time pressure	A busy work environment may hinder the implementation of interventions [24, 25, 27, 29–31]
Shift work and irregular working hours	Shift work and odd working hours may hinder the implementation of interventions [28, 30, 31]
Organizational support to the intervention	Low support to the intervention from management [31] High turnover among managers [18]

## Results

### Study Selection

The search generated 4191 records from PsycInfo, 8316 from Web of Science and 13,569 from PubMed, for a total of 26,076 records (Fig. 1). After removing duplicates, 21,513 records were screened for title and abstract. After the screening, 21,257 records were excluded as they did not meet the eligibility criteria. After title and abstract screening, we assessed 256 articles from which 11 relevant articles were identified and included in our review (Fig. 1). The study characteristics can be seen in Table 2.

### Data Extraction

Of the 11 studies, 3 were qualitative and 8 were mixed methods studies. Three of the included studies were from Australia, three from the United Kingdom, and one each from the United States, Sweden, Denmark, Germany and, Norway. The majority of studies focused on young workers within health-related professions, such as medical trainees, surgery trainees, psychiatry trainees, apprentices in nursing homes. Studies also focused on young workers within supermarkets and apprentices within commercial cookery/hospitality, electronics and construction trade (plumbing, bricklaying, carpentry, and electrician). None of the included studies had their overall focus on identifying barriers and facilitators to the implementation of interventions targeted towards the working conditions for young workers but still included information concerning this.

### Themes Related to Barriers and Facilitators to Implementation

Through the iterative approach described above, we identified seven overall themes that constituted barriers and/or facilitators in the implementation of interventions aimed at improving the working conditions for young workers. An overview of the identified themes (i.e., facilitators and barriers) can be seen in Table 3.

A more thorough explanation and description of the themes can be seen in the text below.

*Fit between the intervention and the organizational context*: The first factor that we identified highlights that the intervention must fit the organizational context to be successfully implemented. According to the reviewed studies, it is important that the intervention is tailored to fit the work tasks and the job incumbents, that the intervention is targeted towards [27]. Moreover, the results from other studies indicate that the perceived relevance of the proposed interventions constitutes an important facilitator for the implementation of interventions for young workers [22, 23, 31]. For instance, the study from Deady et al. [22] showed that the implementation of an app to support the mental health of young workers was supported by the perceived relevance of the app. In a similar vein, Fish and colleagues [24] found that a generic format of a training program to support educational activities in a hospital constituted a significant barrier to its implementation. The results from the scoping review also suggested that it is a facilitating factor when intervention activities are embedded in already existing structures or programmes in the workplace [25]. This supported the implementation of interventions aimed towards young workers, as the intervention was deployed through already existing structures, channels, and/or networks in the workplace. A final facilitating factor, related to the implementation of organizational interventions may be that the person facilitating the intervention has relevant experience within the type of work that the intervention is deployed in [27]. In the study from Kang and colleagues [27], it was considered a clear facilitator that the employees perceived that the intervention was not a generic programme but rather was adapted to the clinical setting in the workplace [27]. Taken together, the identified studies indicate that the fit between the intervention and the various levels of the organization is an important determinant for the success of the implementation of workplace interventions aimed at young workers.

*Organizational support to the intervention:* The second factor that we identified in the scoping review pertains to the organizational support that is required to successfully implement interventions to enhance the work environment and well-being for young workers. First, the results show that support from the top-and middle-level leadership is essential for the implementation of interventions to improve the working conditions for young workers [18, 24, 25]. Moreover, the results show that a lack of leadership support [31] and a high level of turnover among managers [18] constitute a significant barrier in this regard. The study from Sørø et al. [31] also suggests that organizational support from managers and co-workers was an important facilitator for successfully implementing the intervention. This organizational support can imply that the co-workers and managers recognise the intervention activities as legitimate tasks even if the workplace is busy and this implies that it may be ‘easier’ to use working time for intervention activities if this is accepted in the workplace [31]. A final element of organizational support regards the continuous support from union representatives and OSH-representatives to implement interventions aiming at enhancing the work environment and well-being of young workers [18]. This supports the involved managers and workers to keep a long-term focus on implementing the interventions.

*Effort–gain balance:* A third factor that is of importance for the successful implementation of interventions targeting young workers is that it must be accessible [28] and not overly time-consuming [30]—i.e., that there is a balance between the efforts and potential gains in the intervention [32]. The accessibility of interventions formed as training activities can be ensured in two ways. Either by making training activities available online to reduce time spent on travelling to and from the training activities [30] or by making the activities available on more occasions [28]. This may enhance the possibilities of fitting the training or intervention activities into a busy work schedule.

*Employee motivation:* The motivation of employees is a central factor in the implementation of interventions in the workplace as the motivation of employees is central to their participation in and support for the intervention [18, 24, 25, 30]. The study from Gummesson et al. [26] cites the literature when observing that employee motivation is enhanced when employees find that the intervention activities are meaningful and relevant and when the employees understand the rationale behind the intervention. According to Rich et al. [30], the commitment of employees towards an intervention may be strengthened when the organization communicates clear and organizationally relevant goals for the intervention and following the findings from Grüne et al. [25], this is important as a lack of perceived ownership and commitment from the workers had an adverse consequence for the implementation of an intervention in an industrial plant. Finally, the study from Albertsen et al. [18] points out that the appointment of an OSH representative for young workers may enhance the motivation and work environment awareness of young workers.

*Employee involvement:* A fifth factor pertains to the involvement of employees in identifying, designing and implementing interventions targeting young workers [18, 22]. This is an important point as the involvement of employees in these processes may strengthen the fit between the intervention and the organizational needs and, hence, the support from co-workers and managers for the implementation process.

*High workload and time pressure:* As a sixth factor, we found that a busy work environment appears to constitute a significant barrier to the implementation of interventions to improve working conditions and well-being in young workers [24, 25, 27, 29–31]. According to Kang et al. [27], a heavy workload may imply that attendance to programs or interventions may clash with the everyday handling of work tasks. In a similar vein, the study from Sørø et al. [31] found that it may be difficult to find the needed time and space to participate in activities—especially if these activities take place in a location away from the workplace or if there are labour shortages, e.g., due to sickness absence.

*Shift work and irregular working hours:* As a seventh and final factor, we have found that shift work arrangements and irregular working hours may constitute important barriers to the implementation of interventions targeting young workers [28, 30, 31]. According to the study from Luton et al. [28], one way to overcome this barrier may—if possible—be to make the intervention activities available on more than one occasion or by making the activities available online. This may particularly be relevant for training activities.

## Discussion

The study presents results from a scoping review aiming to identify and summarize available research literature on barriers and facilitators that play a role in the implementation of workplace interventions aiming at improving the work environment, safety and health of young workers. Despite our comprehensive search strategy we only identified 11 relevant studies that contained an evaluation of the process of implementing the planned interventions. The 11 studies that we included in this study revealed several factors to be aware of when designing and implementing interventions to support young workers’ safety, work environment and health.

### Discussion of Study Results

In this scoping review, we identified several thematic factors that, respectively, constituted barriers and facilitators. When looking at the facilitating factors, the review showed that a good fit between the intervention and the organization and high levels of organizational support from the organization were conducive to the effective implementation of interventions targeted at young workers. The review also showed that higher levels of employee involvement and motivation as well as a perceived balance between the efforts and potential gains of the intervention also served as facilitating factors in the implementation of the investigated interventions. The findings particularly highlight how organizational support can act as both facilitator and barrier. While strong management support enables successful implementation, low support or high manager turnover can impede intervention efforts and, hence, constitute a barrier. This dual nature emphasizes the critical role of stable, committed leadership in implementing workplace interventions for young workers. Taken together, the presence of these factors in the implementation of the investigated interventions all served as facilitators. It seems appropriate, however, to conceptualize these factors as having a dual nature, because they may indeed constitute barriers if they are absent during the implementation of an intervention targeted towards young workers. These findings are in accordance with the recommendations in the ten Sigtuna Principles focusing on how to design, implement and evaluate organizational interventions for maximum impact [32]. The review findings suggest that employee involvement and motivation are closely linked facilitating factors. When young workers are involved in participatory processes to identify and design interventions, this appears to enhance their motivation to participate and support implementation efforts. This underscores the importance of participatory approaches specifically tailored to engage young workers. However, only two of the studies that were included in this review employed a participatory approach to develop and implement intervention activities [18, 25].

The scoping review also identified two clear barriers that may serve as opposing forces to implementing interventions in workplaces. These two factors pertain to busy work schedules where the targeted workers are exposed to high workloads and high levels of time pressure and also in work environments that are characterized by shift work and/or irregular working hours. On the one hand, these barriers may imply that the possibilities of workers to support the implementation of workplace interventions are severely hampered when the workloads are high and this, on the other hand, is also the case if workers do not attend work at the same time. While both high workload and irregular hours were barriers, the studies suggest potential practical solutions. Online delivery formats and flexible scheduling of intervention activities could help overcome these structural barriers, though this requires organizational commitment to providing such flexibility and resources. This connects back to the importance of organizational support as both a potential facilitator and a barrier. These findings underscore the importance of carefully considering the organizational context and the specific work conditions of young workers when designing and implementing workplace interventions, as failing to account for these factors may hinder the successful adoption and effectiveness of the interventions. The identified need for balance between efforts and gains has practical implications for intervention design. Making activities accessible through online formats or multiple sessions not only addresses time constraints but may also improve the perceived cost-benefits for young workers, potentially increasing participation and implementation success.

Overall, the seven themes that we identified in this scoping review are, with one notable exception, in agreement with the six factors that were presented in the Introduction as being important for the successful implementation of workplace intervention in specific settings [14–16]. The studies included in the present review did not mention the importance of communication in the implementation of workplace interventions aimed at improving working conditions. By contrast, previous studies—not specific for young workers—highlight the importance of effective communication when implementing interventions at the workplace. According to the reviews from Roodbari et al. [14] and Yarker et al. [15], effective communication appears to be an important facilitator when implementing workplace interventions as frequent and systematic communication about the intervention contributes to continuity of efforts. Moreover, communication about working conditions is also considered an important aspect of the psychosocial safety climate in workplaces [33]. Incorporating effective communication strategies into the intervention process, such as regular updates, feedback sessions, and opportunities for all workers regardless of age to provide input, may help contribute to the successful implementation and sustainability of the intervention.

### Implications for Practice and Future Research

When comparing these findings with findings from previous systematic reviews evaluating processes of implementing workplace interventions [14–16], we find similar patterns in the identified facilitators and barriers. This suggests that fundamental facilitators and barriers for workplace interventions are relatively consistent across different worker populations. However, this does not mean that implementing interventions for young workers is identical to implementing interventions for the general workforce. Although the identified barriers and facilitators for interventions targeting young workers are similar to the barriers and facilitators identified in the broader literature [14–16], their relative importance, intensity, and practical relevance may differ when targeting young workers. For example, organizational support and communication strategies may require more attention due to young workers' limited experience with structures and relationships at the workplace. Similarly, participatory approaches may need adaptation to effectively involve young workers who might be less comfortable expressing concerns in established workplace hierarchies. Also, young workers' generally lower awareness of their rights and knowledge about occupational health and safety may influence how interventions are received, understood, and implemented. What might be considered sufficient communication for experienced workers may be inadequate for younger workers who may be lacking in workplace experience. Also, studies report that apprentices often hesitate to engage supervisors or co-workers directly, when experiencing problems in their work environment [8]. The relationship between these implementation factors and intervention effectiveness for young workers remains an important area for investigation. Future comparative studies examining both young and older workers would be valuable in exploring these dynamics further.

As stated in the introduction of this study, young employees are faced with a range of adverse working conditions in terms of increased exposure to occupational accidents, poor safety climate, physically demanding work tasks and chemical substances in the work environment [2–5]. Since research also documents a higher prevalence of depression and anxiety symptoms among young workers in Denmark [3], it is important to be particularly attentive towards the working conditions of young employees to prevent potential long-term consequences of adverse working conditions in the early stages of the work-lives of young employees. One way to improve the work environment of young workers may be to nurture the awareness of young workers towards work environment, safety and health. In this regard, it may be relevant to look into the study from Albertsen et al. [18], which proposes to involve representatives for young workers in the regular meetings in the OSH committee in the workplace. This may, on the one side, increase the attention of the established OSH system in the workplace to the working conditions and health of young workers in the workplace, while simultaneously empowering the young workers in the workplace by giving them a voice in the formal OSH system in the workplace. Furthermore, involving young workers in the OSH meetings may also provide valuable insights into the unique challenges and perspectives of this age group, which can help target effective interventions addressing their specific needs in the workplace.

Research has documented that young workers face significant challenges in their work environment, which can not only compromise their immediate health and well-being but also threaten the long-term sustainability of their work-lives. This underscores the urgent need for early preventive measures in the workplace. Accordingly, previous research highlights the importance of identifying effective and implementable interventions to support young workers' health, safety, and work environment [2, 3, 13].

While prior research has identified key factors for the successful implementation of workplace interventions [14–16], little is known about the specific barriers and facilitators affecting interventions targeting young workers. Our study builds on this knowledge by providing a comprehensive identification and overview of existing research, contributing to a better understanding of the factors essential for designing and implementing effective interventions to improve young workers' work environment, safety, and health.

As also elaborated on in the Strengths and Limitations section, we only identified a limited number of relevant studies, and we found no studies that specifically aimed at identifying barriers or facilitators for implementation of workplace interventions aiming at young workers. This emphasizes the need for future intervention studies focusing on identification and evaluation of facilitators and barriers for implementing interventions for young workers, and not solely on the effectiveness of such interventions.

### Strengths and Limitations

We did not find any studies which explicitly investigated or reported (specifically aiming at investigating or describing), in the title or abstract, barriers and facilitators to the implementation of workplace interventions aiming at improving work environment, safety and health for young workers. However, several articles investigated or described factors that could be interpreted as a barrier or facilitator for the implementation, and were therefore included in the review. As per the pre-registered review-protocol, we aimed to perform a systematic review—with accompanying evidence synthesis—on barriers and facilitators that may impact the implementation of workplace interventions designed to support young workers' health, safety and work environments. Due to the limited number of relevant studies and the fact that we found no studies specifically aimed at identifying barriers or facilitators, we deemed it more relevant and necessary to report the article in the form of a scoping review. Thus, we decided to employ a scoping review approach to identify available literature and summarize key concepts in emerging fields, “especially where an area is complex or has not been reviewed comprehensively before” [34–37].

Acknowledging the likelihood of limited scientific literature specifically addressing young workers in this context, we intentionally designed a broad search strategy with high sensitivity. This was done to ensure we captured potentially relevant literature thus minimizing the risk of overlooking important studies. We did not exclude specific study designs (qualitative, mixed methods, or quantitative) as long as they adhered to the inclusion criteria mentioned above. Thus, we included any study that described barriers or facilitators to the implementation of a workplace intervention among young workers. Still, we encountered a limited number of eligible intervention studies for inclusion, underscoring the need for more research on factors of importance for the successful implementation of workplace-based interventions among young workers. This aligns with our previously published systematic review, which highlighted the limited high-quality research on the effectiveness of workplace interventions for young workers’ safety, environment, and health [13].

Due to the limited research in this area and the absence of intervention studies in the scientific literature specifically aimed at identifying barriers or facilitators for implementing workplace interventions to support young workers' safety, work environment, and health, the overall quality of the included studies is likely not high. In line with this, no quality assessment of the included studies was conducted, as our scoping review was designed to identify and summarize existing literature rather than evaluate the quality of the identified studies. This approach aligns with the scoping review methodology, where a structured quality assessment is not a mandatory requirement [36, 37]. As a result, the review findings and conclusions may be based on studies with potential biases and limitations, which should be considered when interpreting their applicability. This represents a clear limitation of the available evidence and underscores the need for further research in this area.

The gap in research on workplace interventions to support young workers likely exists because research on young individuals often emphasizes education and workforce entry, while their work environment receives comparatively less attention. This likely arises from the prevailing focus on the critical role of education in youth employment, which draws significant attention from policymakers and researchers [38]. Additionally, young workers are a smaller workforce segment, with shorter tenures and less influence, which is why their OSH problems may be overlooked. Workplace improvements often focus on the broader workforce with comparatively less consideration given to the unique challenges faced by young workers as a distinct demographic. On the other hand, comparing with previous studies across age groups, the same basic mechanisms may apply for implementing interventions to young workers as to workers across all age groups.

Young workers represent a diverse group, engaged in various types of work with distinct occupational exposures and varying levels of attachment to the labour market. Thus, not all young workers face the same challenges, and variations according to gender, industry, job type, jurisdictions, and country are likely to also impact how well interventions are implemented along with their effectiveness. Further, different industries and workplaces are likely to exhibit diverse working conditions and working cultures, implying that both implementation factors and effectiveness of interventions for young workers can vary significantly. This emphasizes the necessity of nuanced approaches to enhancing the working environment for young people in the workforce. Thus, the limited amount of relevant studies in the scientific literature which do not allow for a stratified and comparative approach may constitute a limitation to our review. Future studies are needed to minimize the risks of overlooking key elements in the understanding of who benefits from these interventions and how they could be improved.

The majority of studies focused on young workers within health-related professions, such as medical trainees, surgery trainees, psychiatry trainees, apprentices in nursing homes. Studies also focused on young workers within supermarkets and apprentices within commercial cookery/hospitality, electronics and construction trade (plumbing, bricklaying, carpentry, and electrician) (see also Table 2). Overall, it seems that the findings of the present study primarily reflect barriers and facilitators to implementation for young workers within health-related professions. Future studies should focus on interventions aiming at identifying and evaluating factors of importance for implementation among young workers in non-health-related professions.

The included studies reflects a somewhat geographical imbalance since the 11 included studies were from seven different countries (Australia, United Kingdom, United States, Sweden, Denmark, Germany and, Norway). Despite a somewhat international perspective, it limits the generalizability of the present study results.

This review compiles existing knowledge on barriers and facilitators to implementation of interventions among young worker (irrespective of education, industry, positions, experience, labour market, etc.). To include young workers entering the workforce later due to longer education, we consider intervention studies up to age 29, an approach used in both Danish and international studies [2, 3, 13]. However, while young workers often overlap with those who are new to the workforce, not all young workers are inexperienced, and not all new workers are young. Many industries have mid-career workers transitioning into new roles, immigrants entering the workforce, or individuals switching careers later in life—all of whom may face similar barriers and facilitators when implementing workplace interventions. With the studies focus on young workers (i.e., age) rather than experience, it may overlook important insights into how interventions function across different career stages.

The current review exclusively relies on studies in English which could introduce bias by potentially excluding valuable evidence from other languages in the domain of work environment and health. Hence, the possibility of language restriction bias in this study cannot be overlooked.

## Conclusions

This scoping review identifies 11 studies containing information on factors to be aware of when designing and implementing interventions to support young workers’ safety, work environment and health. Based on the identified studies, seven primary themes related to facilitators and barriers to implementation emerged from our analysis: Facilitators: fit the organizational context, organizational support, balance between efforts and gains, employee motivation, and employee involvement; Barriers: high workload and time pressure, shift work and irregular working hours. The results emphasize a need for studies evaluating factors of importance for the successful implementation of workplace-based interventions among young workers.

## Supplementary Information

Below is the link to the electronic supplementary material.

Supplementary file1 (DOCX 34 KB)

## Data Availability

The data that support the findings of this review will be available from the corresponding author upon reasonable request.
